# Nonlinear dynamics of in-plane ring resonator for mass sensing

**DOI:** 10.1007/s00542-025-05960-8

**Published:** 2025-10-21

**Authors:** Saber Azizi, Hadi Madinei, Hamed Haddad Khodaparast, Peter Steeneken, Mohammad I. Younis, Ghader Rezazadeh

**Affiliations:** 1 Aerospace department, Faculty of Science and Engineering, Swansea University, Swansea, UK; 2 Department of Precision and Microsystems Engineering, Delft University of Technology, Delft, Netherlands; 3 Department of Mechanical Engineering, State University of New York, Binghamton, USA; 4 Centre for Materials Technologies, Skolkovo Institute of Science and Technology, Moscow, Russia; 5 Mechanical Engineering Department, Faculty of Engineering, Urmia University, 11km Sero Road, Urmia, 16557153 Iran

## Abstract

Mass sensing using MEMS is crucial for detecting minute changes in mass with high sensitivity, enabling applications in environmental monitoring, medical diagnostics, and chemical detection. However, fluid damping in these environments is relatively high and can lead to reduction of the quality factor and sensitivity of these sensors. In this paper, we present a rotating ring resonator for mass sensing applications and investigate its nonlinear dynamics and bifurcation. The ring is supported by four slender beams and subjected to rotational base excitation. The shift in the nonlinear bifurcation point on the frequency response curve is used for mass sensing, which is significant because the device exhibits multiple nonlinear bifurcation points. The structure is designed and modelled to vibrate in a rotational in-plane mode, to provide lower damping and higher quality factor compared to cantilever-based mass sensors that operate in a translational out-of-plane mode. Moreover, the structure exhibits nonlinear resonance zones within the super harmonic regime, enabling mass detection at a particular fraction of the primary resonance zone. At lower excitation amplitudes, the linear response dominates, and the device also allows mass detection in the linear regime via resonance frequency shifts.

## Introduction

Over the past decades, there has been a steadily growing demand for the application of microelectromechanical systems (MEMS) (Pasquale and Somà 2010; Azizi et al. 2023; Zamanzadeh et al. 2020). MEMS sensors and actuators benefit from low weight, low fabrication costs, and high sensitivity, which are critically important in high-tech biomedical applications (Yaqoob et al. 2022). Among MEMS sensors and actuators, mass sensors have garnered significant attention for their ability to detect the masses of viruses and biomarkers for disease detection applications (Alneamy and Ouakad 2023; Jingjing, et al. 2016; Alvarez and Lechuga 2010). Measuring extremely small masses in biomedical contexts, such as viruses, bacteria, biomolecules, DNA, or proteins, has always posed significant challenges (Baguet et al. 2019; Chauhan and Ansari 2021; Mohammad 2011; Katzis et al. 2022; Hashoul and Haick 2019). Various sensing mechanisms have been utilised so far for the capturing of the bio masses which include resonance frequency shifts (Park et al. 2012; Chellasivalingam et al. 2020; Joshi et al. 2019), bifurcation based switching (Azizi et al. 2023; Alneamy and Ouakad 2023; Meesala et al. 2020; Nayfeh et al. 2010; Kumar et al. 2011; Yuksel et al. 2019) and symmetry-breaking (Baguet et al. 2019; Chellasivalingam et al. 2020). In terms of sensing mechanisms, the most commonly used ones include piezoelectric (Chellasivalingam et al. 2020; Joshi et al. 2019; Azizi et al. 2017; Kumar, et al. 2011; Xu and Yan 2024; Toledo et al. 2019), electrostatic (Baguet et al. 2019; Elliott et al. 2017; Botamanenko et al. 2023), and magnetic detection (Jafari et al. 2017; Timurdogan et al. 2011). Though bifurcation based sensors have been addressed in the literature to measure mass there are some complications regarding the application of phase-locked loops (PLL), to address this issue, Yuksel et al. (2019) introduced a trajectory-locked loop (TLL) architecture to enable stable mass sensing in the nonlinear regime of Duffing resonators. In this method, the system avoids locking to a fixed phase instead, it continuously circulates within the hysteresis window, alternating between the jump frequencies ${f}_{up}$ and ${f}_{down}$. This approach allows for rapid and sensitive tracking of frequency shifts induced by single particle events, even beyond the linear dynamic range of the sensor.

MEMS mass sensors have been utilized in the diagnosis of various diseases to date. Timurdogan et al. applied resonant microcantilever arrays (Timurdogan et al. 2011), functionalised with Hepatitis antibodies. They detected both Hepatitis *A* and Hepatitis *C* antigens. Shafiee et al. (Shafiee et al. 2013), detected HIV virus through label-free electrical sensing of viral nano-lysate. Chen et al. (Li et al. 2019) developed a microcantilever array biosensor for simultaneously measuring two biomarkers carcinoembryonic antigen (CEA) and $\alpha$-fetoprotein (AFP) by means of an optical readout technique. One of the challenges of cantilever beam sensors is that their stiffness is influenced by the thickness and the position of the mass of the antigen or biomarker deposited on the beam. This absorption-induced stiffness can lead to errors in mass detection (Jingjing et al. 2016; Alvarez and Lechuga 2010; Johnson and Mutharasan 2012; Lee et al. 2004). Wang et al. (Jingjing, et al. 2016) developed a cantilever based sensor for the detection of the biomarkers of liver cancer with various concentrations of AFP. They reported a huge frequency shift of 830 Hz due to absorption induced stiffness which was two orders of magnitude larger than the theoretical calculations. To address the challenge of absorption induced stiffness, they designed a micro-cavity at the free end of the cantilever beams for local antibody immobilization and as a result reduced the absorption induced stiffness of the system. A similar approach was taken by Wang et al. (2015), to reduce the absorption induced stiffness of a the arrays of cantilever beams. Stachiv al. (2022) reported on the challenges posed by size and absorption-induced stiffness in cantilever-based sensors. In another study Stachiv et al. (2020) demonstrated that for heavy analytes (> *MDa*), conventional multimode frequency shift methods yield inaccurate mass estimates. They proposed a technique based on monitoring Q-factor changes in air, eliminating the need to resolve analyte position or stiffness. Their method showed highest sensitivity using lateral modes and enabled accurate mass detection of large biomolecules and cells. Cantilever-based mass sensors have also been used to measure volatile organic compounds (VOCs) in exhaled breath (Kurmendra and R. Kumar 2019; Gupta et al. 2018). However, the challenges of dependency and absorption-induced stiffness remain significant. Biosensing applications require the sensor to be used in air and liquid environment (Johnson and Mutharasan 2012). Another challenge in the application of cantilever-based bio medical mass sensors is the high damping ratio and accordingly low quality factor which is mainly due to the out of plane motion of the cantilever beam (Timurdogan et al. 2011; Azizi et al. 2012; Hansen and Thundat 2005), especially position when it is operated in liquid; Various methods have been proposed to address the challenge of high damping ratio involved in bio mass detection; these include the so called “dip & dry” method (Timurdogan et al. 2011) which brings about other implications working with activated microbes, wetting, and change in stiffness due to surface adhesion (Gfeller et al. 2005). Lee et al. (2011) designed microchannel inside the cantilever beams to allow the liquid to pass through; although the method was a novel approach but was able to pass very small sample volumes. Olcum et al. (2014) designed and fabricated a nano resonator based on cantilever structure to detect self-assembled DNA nano particles. Some investigations have been carried out to operate cantilever-based mass sensors in higher resonant modes to decrease the associated damping ratio. Higher modes result in increased stiffness, reduced damping ratios, and, consequently, a higher quality factor (Johnson and Mutharasan 2012; Maraldo and Mutharasan 2007);

As discussed, despite their popularity in biomass sensing, cantilever-based biomass sensors face two main challenges: absorption-induced stiffness and high damping ratios due to out-of-plane motion. To reduce damping, and increase Q-factor, it is beneficial to minimize the amount of liquid that needs to be displaced. To achieve this operation mode, we propose a rotational ring biomass sensor featuring a ring-based structure supported by four clamped–clamped microbeams. This model is expected to benefit from a significantly low damping ratio due to the in-plane nature of the motion. The advantage of the middle ring over the disc is that it minimizes the impact of position-dependent added mass on the dynamics of the central disc. The biomass is assumed to be deposited on the central ring, which is relatively thin in comparison to the overall structure. The model minimises absorption-induced stiffness and exhibits significantly reduced damping due to in-plane motion (Azizi et al. 2012). The frequency response curves are derived using the continuation technique, and the bifurcation points are identified. To verify the continuation approach, the frequency response curves in the vicinity of the first two primary resonances are derived based on multiple time scales method. The system is designed to operate near the catastrophic bifurcation points (Nayfeh and Balachandran 2008), such that biomass deposition triggers the bifurcation, enabling biomass detection. The designed system targets measuring bio masses in the order of picograms which is ideal for the detection of the biomarkers of variety of cancers including liver (AFP), Prostate (PSA) and Lung cancer (CA-125).

## Modelling

**Fig. 1:**
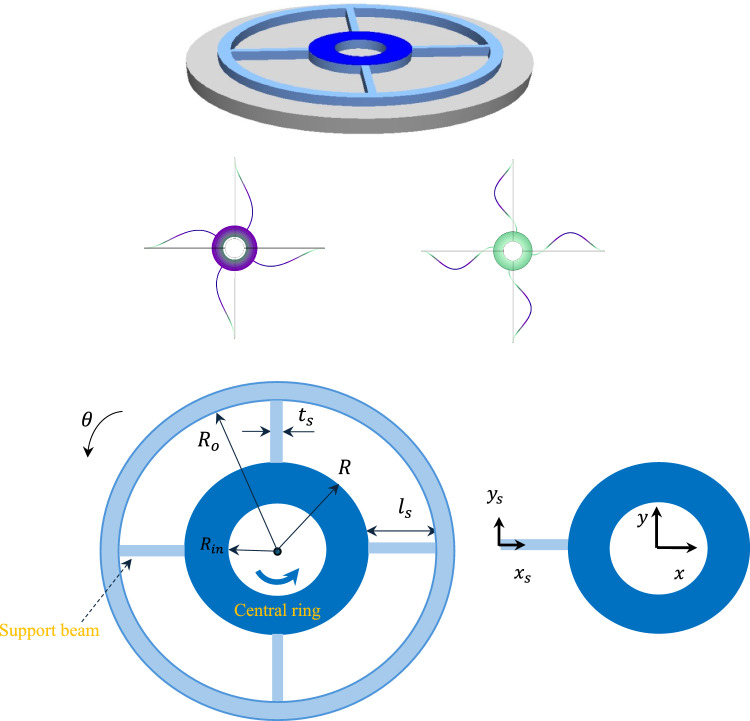
Schematics of the ring and the support beam subjected to base excitation, with the first two mode shapes included (by COMSOL)

The model we have investigated, benefits from in-plane motion, which helps to address the significant damping typically observed in cantilever-based mass sensors due to their out-of-plane motion. The schematics of the model which consists of a ring-based mass along with the supporting beams are illustrated in Fig. 1. The supporting beams, which contribute torsional stiffness to the central ring (as shown in the first two mode shapes in Fig. 1), are connected at one end to the central ring and at the other end to the fully fixed substrate. The outer and inner radii of the central ring are denoted by $R$ and ${R}_{i}$ respectively. The supporting beams have length ${l}_{s}$, thickness $h$, and width of ${t}_{s}$. The substrate is assumed to be rigid and is mounted on top of a motion-controlled base, which applies harmonic excitation in the form of $\theta \left(t\right)={\theta }_{0}\text{sin}(\omega t)$. The coordinate system ${x}_{s}-{y}_{s}$ is attached to the left end of the support beam. The material has a density $\rho$ and Young’s modulus $E$. The coordinate system *x–y* is attached to the centre of the ring and rotates with it.

Considering inertial coordinate system *x–y* with unit vectors (***i-j***) which is attached to the centre of the ring, the position vector ${\overrightarrow{R}}_{p}$ of a point ‘*p*’ on the support beam can be expressed in the form of Eq. (1).

1 $${\overrightarrow{R}}_{p}=\left(-{R}_{o}+{x}_{s}\right){\varvec{i}}+{w}_{s}{\varvec{j}}$$

Considering the harmonic base excitation $\theta \left(t\right)$, the velocity of point ‘*p*’ is in the following form (Azizi et al. 2012):

2 $${\dot{\overrightarrow{R}}}_{p}=-{\dot{\theta }w}_{s}{\varvec{i}}+\left({\dot{w}}_{s}+\dot{\theta }\left(-{R}_{o}+{x}_{s}\right)\right){\varvec{j}}$$

Considering Eq. (2) The kinetic energy of the system can be expressed in the following form (Azizi et al. 2023; Azizi et al. 2017; Azizi et al. 2022):

3 $$ \begin{array}{*{20}l} {T = \frac{1}{2}\left( {\rho A} \right)_{s} n_{s} \smallint _{0}^{{l_{s} }} \left( {\left( { - \dot{\theta }w_{s} } \right)^{2} + \left( {\dot{w}_{s} + \dot{\theta }\left( { - R_{o} + x_{s} } \right)} \right)^{2} } \right)dx_{s} } \hfill \\ {\quad + \frac{1}{2}I_{D} \left( {\dot{\varphi } + \dot{\theta }} \right)^{2} } \hfill \\ \end{array} $$

Where ${I}_{D}$ represents the mass moment of inertia of the disc and the added mass about the centre of the disc, $\dot{\varphi }$ denotes the angular velocity of the disc relative to the base and ${n}_{s}$ is the number of support beams. Considering the assumption of the stretching effect and neglecting the longitudinal inertia (Nayfeh and P.F.P., 2004; Azizi et al. 2014), The potential energy of the support beams is expressed in the following form:

4 $$U=\frac{1}{2}{(EI)}_{s}{n}_{s}{\int }_{0}^{{l}_{s}}{{w}_{s}^{{\prime}{\prime}}}^{2}d{x}_{s}+\frac{EA}{8{l}_{s}}{n}_{s}{\left({\int }_{0}^{{l}_{s}}{{w}_{s}{\prime}}^{2}d{x}_{s}\right)}^{2}$$

Where, *I* and *A* are the second moment of inertia about the neutral axis and the cross-sectional area of the support beams. Assuming the solution to be in the form of ${w}_{s}\left({x}_{s},t\right)={\sum }_{i}^{n}{q}_{i}\left(t\right){\psi }_{{s}_{i}}\left({x}_{s}\right)$ (Rashidi et al. 2024, 2023), where ${q}_{i}\left(t\right)$ and ${\psi }_{{s}_{i}}\left({x}_{s}\right)$ are the generalised coordinates and the mode shapes which satisfy the boundary conditions associated with the support beams, the kinetic and the potential energies reduce to:

5 $$ \begin{array}{*{20}l} {T = \frac{1}{2}\left( {\rho A} \right)_{s} n_{s} \left( {\dot{\theta }^{2} \sum _{{i = 1}}^{n} \sum _{{j = 1}}^{n} q_{i} \left( t \right)q_{j} \left( t \right)\smallint _{0}^{{l_{s} }} \psi _{{s_{i} }} (x_{s} )\psi _{{s_{j} }} (x_{s} )dx_{s} } \right.} \hfill \\ {\quad + \sum _{{i = 1}}^{n} \sum _{{j = 1}}^{n} \dot{q}_{i} \left( t \right)\dot{q}_{j} \left( t \right)\smallint _{0}^{{l_{s} }} \psi _{{s_{i} }} \left( {x_{s} } \right)\psi _{{s_{j} }} \left( {x_{s} } \right)dx_{s} } \hfill \\ {\quad + \dot{\theta }^{2} \smallint _{0}^{{l_{s} }} \left( {R_{o} - x_{s} } \right)^{2} dx_{s} } \hfill \\ {\left. {\quad - 2\dot{\theta }\sum _{{i = 1}}^{n} \dot{q}_{i} \left( t \right)\smallint _{0}^{{l_{s} }} \left( {R_{o} - x_{s} } \right)\psi _{{s_{i} }} (x_{s} )dx_{s} } \right)} \hfill \\ {\quad + \frac{1}{2}I_{D} \left( {\sum\limits_{{i = 1}}^{n} {\sum\limits_{{j = 1}}^{n} {\dot{q}_{i} } } \left( t \right)\dot{q}_{j} \left( t \right)\psi \prime _{{s_{i} }} \left( {l_{s} } \right)\psi \prime _{{s_{j} }} \left( {l_{s} } \right) + \dot{\theta }^{2} } \right.} \hfill \\ {\left. {\quad + 2\dot{\theta }\sum\limits_{{i = 1}}^{n} {\dot{q}_{i} } \left( t \right)} \right)} \hfill \\ \end{array} $$

$$ \begin{array}{*{20}l} {U = \frac{1}{2}(EI)_{s} n_{s} \left( {\sum\limits_{{i = 1}}^{n} {\sum\limits_{{j = 1}}^{n} {q_{i} } } \left( t \right)q_{j} \left( t \right)\int\limits_{0}^{{l_{s} }} {\psi _{{s_{i} }}^{{\prime \prime }} \left( {x_{s} } \right)\psi _{{s_{j} }}^{{\prime \prime }} \left( {x_{s} } \right)dx_{s} } } \right)} \hfill \\ {\quad + \frac{{EA}}{{8l_{s} }}n_{s} \left( {\sum\limits_{{i = 1}}^{n} {\sum\limits_{{j = 1}}^{n} {\sum\limits_{{k = 1}}^{n} {\sum\limits_{{o = 1}}^{n} {q_{i} } } } } \left( t \right)q_{j} \left( t \right)q_{k} \left( t \right)q_{o} \left( t \right)} \right.} \hfill \\ {\left. {\quad \times \int\limits_{0}^{{l_{s} }} {\psi _{{s_{i} }}^{\prime } \left( {x_{s} } \right)\psi _{{s_{j} }}^{\prime } \left( {x_{s} } \right)dx_{s} } {\text{ }}\int\limits_{0}^{{l_{s} }} {\psi _{{s_{k} }}^{\prime } \left( {x_{s} } \right)\psi _{{s_{o} }}^{\prime } \left( {x_{s} } \right)dx_{s} } } \right)} \hfill \\ \end{array} $$

The derivation process of the associated shape functions is given in appendix 1. In Eq. (5) the angular velocity of the central ring with respect to the base ($\dot{\varphi }$), has been approximated by $\sum_{i=1}^{n}{{\psi }{\prime}}_{{s}_{i}}\left({l}_{s}\right){\dot{q}}_{i}\left(t\right)$ which is the gradient of the support beams at ${x}_{s}={l}_{s}$.

To account for the effect of the dissipation on the motion equations, the Rayleigh’s dissipation function is defined as follows (Mohammad and Y., 2011):

6 $${R}_{D}=\frac{1}{2}{c}_{s}{n}_{s}{\int }_{0}^{{l}_{s}}{\left(\frac{\partial {w}_{s}}{\partial t}\right)}^{2}d{x}_{s}$$

Where, ${c}_{s}$ is the damping coefficient per unit length of the support beam. Considering the non-dimensionalising parameters ${T}_{t}$, $g$ and $\Theta$ the non-dimensional parameters $\widehat{t}$, ${\widehat{w}}_{s}$ and $\widehat{\theta }$ are defined in the following form.

7 $$\widehat{t}=\frac{t}{{T}_{t}} {\widehat{w}}_{s}=\frac{{w}_{s}}{g} \widehat{\theta }=\frac{\theta }{\Theta }$$

For simplicity the over hats have been removed in the rest of the paper. Introducing Eq. (7) to the Lagrangian function ($L=T-U$) (Azizi et al. 2023; Firoozy et al. 2017), and considering the contribution of the first two modes in the solution, the equations of the motion reduce to:

$$ \begin{array}{*{20}l} {M_{1} \ddot{q}_{1} + M_{2} \ddot{q}_{2} + Kl_{1} q_{1} + Kl_{2} q_{2} + Kn_{1} q_{1} ^{3} + Kn_{2} q_{2} ^{3} } \hfill \\ {\quad \quad + Kg_{1} q_{1} q_{2} ^{2} + Kg_{2} q_{2} q_{1} ^{2} + C_{1} \dot{q}_{1} + C_{2} \dot{q}_{2} = F_{1} } \hfill \\ \end{array} $$

8 $$ \begin{array}{*{20}l} {M_{2} \ddot{q}_{1} + M_{3} \ddot{q}_{2} + Kl_{2} q_{1} + Kl_{3} q_{2} + Kn_{3} q_{1} ^{3} + Kn_{4} q_{2} ^{3} } \hfill \\ {\quad \quad + Kg_{3} q_{1} q_{2} ^{2} + Kg_{4} q_{2} q_{1} ^{2} + C_{2} \dot{q}_{1} + C_{3} \dot{q}_{2} = F_{2} } \hfill \\ \end{array} $$

Where constants in Eq. (8) have been introduced in Appendix 2.

To apply multiple time scales (MTS) as a well-known perturbation based method, the bookkeeping parameter $\varepsilon$ is introduced to keep track of the order of the nonlinear terms in the motion equations (Nayfeh 1981); this has been carried out as follows:

$$ \begin{array}{*{20}l} {M_{1} \ddot{q}_{1} + M_{2} \ddot{q}_{2} + Kl_{1} q_{1} + Kl_{2} q_{2} } \hfill \\ {\quad + \varepsilon \left( {Kn_{1} q_{1} ^{3} + Kn_{2} q_{2} ^{3} + Kg_{1} q_{1} q_{2} ^{2} + Kg_{2} q_{2} q_{1} ^{2} + C_{1} \dot{q}_{1} + C_{2} \dot{q}_{2} } \right) = \varepsilon F_{1} } \hfill \\ \end{array} $$

9 $$ \begin{array}{*{20}l} {M_{2} \ddot{q}_{1} + M_{3} \ddot{q}_{2} + Kl_{2} q_{1} + Kl_{3} q_{2} + \varepsilon \left( {Kn_{3} q_{1}^{3} + Kn_{4} q_{2}^{3} } \right.} \hfill \\ {\quad \left. { + Kg_{3} q_{1} q_{2}^{2} + Kg_{4} q_{2} q_{1}^{2} + C_{2} \dot{q}_{1} + C_{3} \dot{q}_{2} } \right) = \varepsilon F_{2} } \hfill \\ \end{array} $$

To enable the investigation of primary resonance, the excitation forces must be of an order of magnitude less than those in the linear problem; otherwise, they will not appear in the solvability condition of the first order. This does not imply that primary resonance will not occur otherwise, but it guarantees that the conditions of the appearance of primary resonance are met. Assuming the asymptotic solution for ${q}_{1}$ and ${q}_{2}$ to be in the following form:

$${q}_{1}\left({T}_{0},{T}_{1}\right)={u}_{0}\left({T}_{0},{T}_{1}\right)+\varepsilon {u}_{1}\left({T}_{0},{T}_{1}\right)+\text{\rm O}({\varepsilon }^{2})$$

10 $${q}_{2}\left({T}_{0},{T}_{1}\right)={v}_{0}\left({T}_{0},{T}_{1}\right)+\varepsilon {v}_{1}\left({T}_{0},{T}_{1}\right)+{\rm O}({\varepsilon }^{2})$$

where, ${T}_{0}=t$, ${T}_{1}=\varepsilon t$ are fast and slow time scales respectively. Substituting the asymptotic solutions in Eq. (10), considering ${D}_{n}=\frac{\partial }{\partial {T}_{n}}$, and equating coefficients of like powers of $\varepsilon$ we conclude:

11 $${\varepsilon }^{0}:{M}_{1}{D}_{0}^{2}{u}_{0}+{M}_{2}{D}_{0}^{2}{v}_{0}+{Kl}_{1}{u}_{0}+{Kl}_{2}{v}_{0}=0$$

$${M}_{2}{D}_{0}^{2}{u}_{0}+{M}_{3}{D}_{0}^{2}{v}_{0}+{Kl}_{2}{u}_{0}+{Kl}_{3}{v}_{0}=0$$

$$ \begin{array}{*{20}l} {\varepsilon ^{1} :M_{1} D_{0}^{2} u_{1} + M_{2} D_{0}^{2} v_{1} + Kl_{1} u_{1} + Kl_{2} v_{1} } \hfill \\ {\quad = - 2M_{1} D_{0} D_{1} u_{0} - 2M_{2} D_{0} D_{1} v_{0} - Kn_{1} u_{0} ^{3} - Kn_{2} v_{0} ^{3} - Kg_{1} u_{0} v_{0} ^{2} } \hfill \\ {\quad - Kg_{2} v_{0} u_{0} ^{2} - C_{1} \dot{u}_{1} - C_{2} \dot{v}_{1} + F_{1} } \hfill \\ \end{array} $$

$$ \begin{array}{*{20}l} {M_{2} D_{0}^{2} u_{1} + M_{3} D_{0}^{2} v_{1} + Kl_{2} u_{1} + Kl_{3} v_{1} } \hfill \\ {\quad = - 2M_{2} D_{0} D_{1} u_{0} - 2M_{3} D_{0} D_{1} v_{0} - Kn_{3} u_{0} ^{3} - Kn_{4} v_{0} ^{3} } \hfill \\ {\quad - Kg_{3} u_{0} v_{0} ^{2} - Kg_{4} v_{0} u_{0} ^{2} - C_{2} \dot{u}_{1} - C_{3} \dot{v}_{1} + F_{2} } \hfill \\ \end{array} $$

The solution to order ${\varepsilon }^{0}$ Eq. (12) is expressed in the following form:

12 $$\left\{\begin{array}{c}{u}_{0}\left({T}_{0},{T}_{1}\right)\\ {v}_{0}\left({T}_{0},{T}_{1}\right)\end{array}\right\}=\left[\begin{array}{cc}1& -0.19\\ 0.04& 0.98\end{array}\right]\left\{\begin{array}{c}A\left({T}_{1}\right){e}^{i{{\omega }_{n}}_{1}{T}_{0}}\\ B\left({T}_{1}\right){e}^{i{{\omega }_{n}}_{2}{T}_{0}}\end{array}\right\}+CC$$

In Eq. (12), the modal matrix is derived from the coupled linear free vibration problem. $A\left({T}_{1}\right)$ and $B\left({T}_{1}\right)$ are undetermined at this level of approximation; they are determined at the next level by imposing the solvability condition. Substituting Eq. (12), in the order ${\varepsilon }^{1}$ Eq. (11), and expressing the forcing terms in ${F}_{1}={f}_{1}\frac{1}{2}\left({e}^{i\Omega {T}_{0}}+{e}^{-i\Omega {T}_{0}}\right)$, ${F}_{2}={f}_{2}\frac{1}{2}\left({e}^{i\Omega {T}_{0}}+{e}^{-i\Omega {T}_{0}}\right)$ and seeking a particular solution free of secular terms in the form of:

13 $$\left\{\begin{array}{c}{u}_{0}\\ {v}_{0}\end{array}\right\}=\left[\begin{array}{cc}{Q}_{1}({T}_{1})& {Q}_{2}({T}_{1})\\ {P}_{1}({T}_{1})& {P}_{2}({T}_{1})\end{array}\right]\left\{\begin{array}{c}{e}^{i{{\omega }_{n}}_{1}{T}_{0}}\\ {e}^{i{{\omega }_{n}}_{2}{T}_{0}}\end{array}\right\}+CC$$

Introducing Eq. (13), to Eq. (12), we obtain:

$$ \begin{array}{*{20}l} {\left[ {\left( { - M_{1} \omega _{{n_{1}^{2} }} + Kl_{1} } \right)Q_{1} + \left( { - M_{2} \omega _{{n_{1}^{2} }} + Kl_{2} } \right)P_{1} } \right]e^{{i\omega _{{n_{1} }} T_{0} }} } \hfill \\ {\quad + \left[ {\left( { - M_{1} \omega _{{n_{2}^{2} }} + Kl_{1} } \right)Q_{2} + \left( { - M_{2} \omega _{{n_{2}^{2} }} + Kl_{2} } \right)P_{2} } \right]e^{{i\omega _{{n_{2} }} T_{0} }} } \hfill \\ {\quad = \chi _{{11}} e^{{i\omega _{{n_{1} }} T_{0} }} + \chi _{{12}} e^{{i\omega _{{n_{2} }} T_{0} }} } \hfill \\ \end{array} $$

14 $$ \begin{array}{*{20}l} {\left[ {\left( { - M_{2} \omega _{{n_{1}^{2} }} + Kl_{2} } \right)Q_{1} + \left( { - M_{3} \omega _{{n_{1}^{2} }} + Kl_{3} } \right)P_{1} } \right]e^{{i\omega _{{n_{1} }} T_{0} }} } \hfill \\ {\quad + \left[ {\left( { - M_{2} \omega _{{n_{2}^{2} }} + Kl_{2} } \right)Q_{2} + \left( { - M_{3} \omega _{{n_{2}^{2} }} + Kl_{3} } \right)P_{2} } \right]e^{{i\omega _{{n_{2} }} T_{0} }} } \hfill \\ {\quad = \chi _{{21}} e^{{i\omega _{{n_{1} }} T_{0} }} + \chi _{{22}} e^{{i\omega _{{n_{2} }} T_{0} }} } \hfill \\ \end{array} $$

By equating the coefficients of each of ${e}^{i{{\omega }_{n}}_{1}{T}_{0}}$ and ${e}^{i{{\omega }_{n}}_{2}{T}_{0}}$ on both sides, we derive two sets of two inhomogeneous algebraic equations for ${Q}_{1}$ $,{P}_{1}$ and${Q}_{2}$,${P}_{2}$. As the homogenous part of the equations have a nontrivial solution, the solvability condition can be expressed as:

15 $$\left|\begin{array}{cc}-{M}_{1}{{{\omega }_{n}}_{1}}^{2}+{Kl}_{1}& {\chi }_{11}\\ -{M}_{2}{{{\omega }_{n}}_{1}}^{2}+{Kl}_{2}& {\chi }_{21}\end{array}\right|=0 and \left|\begin{array}{cc}-{M}_{1}{{{\omega }_{n}}_{2}}^{2}+{Kl}_{1}& {\chi }_{12}\\ -{M}_{2}{{{\omega }_{n}}_{2}}^{2}+{Kl}_{2}& {\chi }_{22}\end{array}\right|=0$$

To describe the closeness of the excitation frequency $\Omega$ to the natural frequencies, the detuning parameter has been defined as, $\Omega ={\omega }_{n}+\varepsilon \sigma$ where $\sigma$ represents the deviation of the excitation frequency from the natural frequency, assuming $A\left({T}_{1}\right)=\frac{1}{2}{a}_{1}({T}_{1}){e}^{i{b}_{1}({T}_{1})}$ and $B\left({T}_{1}\right)=\frac{1}{2}{a}_{2}({T}_{1}){e}^{i{b}_{2}({T}_{1})}$ and applying some simplifications the frequency response curves in the vicinity of the first two primary resonances are obtained.

## Results and discussions

The geometric and mechanical properties of the studied model are given in Table 1.

**Table 1:** Mechanical and geometrical properties of the ring and the support beams

Parameter	Value
${l}_{s}$	150 μm
${R}_{o}$	190 μm
$R$	40 μm
${R}_{in}$	20 μm
$h$	20 μm
$E$	112.4 GPa
${t}_{s}$	2 μm
${n}_{s}$	4
$\rho$	2330 kg/m^3^

**Fig. 2:**
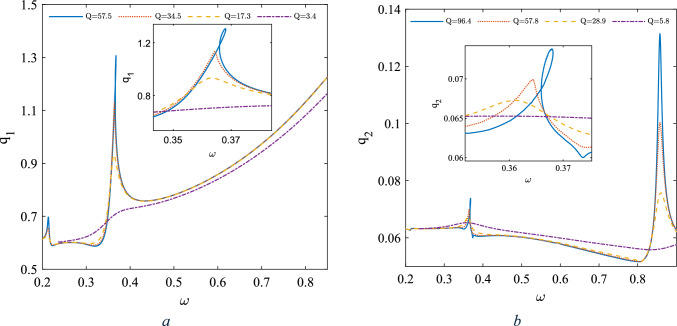
The frequency response curve in the super harmonic resonance zone, **a** amplitude of first mode, **b** amplitude of the second mode $g={10}^{-6} m , {\theta }_{0}=\frac{\pi }{10} rad$

In this analysis, we consider four distinct scenarios, each within the underdamped regime for the damping coefficient ${c}_{s}$.These scenarios are characterized by four different quality factors corresponding to the first two modes, as illustrated in Fig. 2. Due to the nonlinear stiffness terms, sweeping the excitation frequency $\omega$ in the super harmonic regime reveals various resonance zones. The locations of these resonance zones on the frequency axis depend on both the amplitude of the base excitation and the quality factor. The frequency response curves corresponding to the super harmonic regime associated with the first two modes are illustrated below. It is worth mentioning that the amplitude of the base excitation has been assumed to vary i.e. ($\frac{{\theta }_{0}}{{\omega }^{2}}$) such that by increasing the excitation frequency the acceleration $\ddot{\theta }$ amplitude remains constant.

**Fig. 3:**
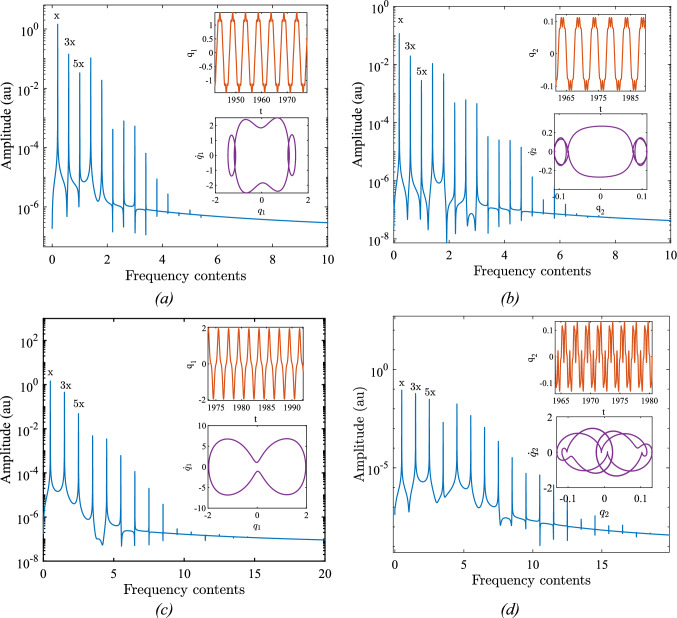
Frequency contents of the response for two different cases: **a**, **b**: $\upomega =0.2$ **c**, **d**: $\upomega =0.5,\text{ g}={10}^{-6}\text{ m }, {\uptheta }_{0}=\frac{\uppi }{10}\text{ rad}$

Due to the sources of nonlinearity, prior to the primary resonance, the system exhibits two super harmoni resonaces one in the vicinity of $\omega =0.2$ (equal to $\Omega =$ 35.6 kHz) and another one in the vicinity of $\omega =0.35$ (equal to $\Omega =$ 62.4 kHz). It is noteworthy that these resonance zones are substantial because they exhibit high-amplitude motion, which is particularly significant in low-frequency resonators. This is crucial in the design of low-frequency MEMS resonators, as these devices are inherently associated with high resonance frequencies. While high resonance frequencies offer enhanced sensitivity, they also come with certain drawbacks that must be considered. In order to investigate the source of each superharmonic resonance zone, we have considered two frequencies, ω = 0.2 and ω = 0.5 (equal to $\Omega =$ 89.12 kHz), both below the primary resonance. Figure 3 depicts the frequency content of both modes for each individual case, with the associated steady-state time response and the phase plane indicated as insets ($g={10}^{-6} \text{m} , {\theta }_{0}=\frac{\pi }{10}\text{ rad}$).

As illustrated, assuming the excitation frequency in both cases to be x, the frequency content of the response includes odd multiples of the excitation frequency, i.e., 3x, 5x, 7x, and so on. This means that the system may be assumed as a function which gives off the odd multiples of the excitation frequency in the response and according any odd fraction i.e. $\frac{{\omega }_{n}}{x}$ of the primary resonance can be assummed as a superharmonic resonance zone. Given that the first primary resonance is in the vicinity of $\omega =1.0$, the presence of the term 5 × maps the excitation frequency to the primary resonance. Consequently, a superharmonic resonance zone appears in the vicinity of $\frac{{\omega }_{n}}{5}$, which indirectly excites the primary resonance and is accordingly known as a superharmonic secondary resonance. The same rational can be applied to justify the presence of the remaining superharmonic resonance zones which are due to the 5x, 7 × and so on.

**Fig. 4:**
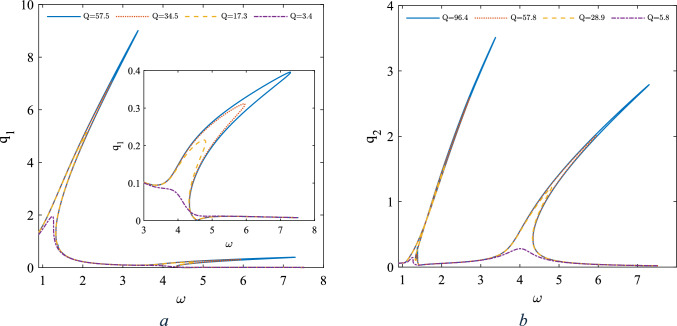
The frequency response curve in the vicinity of the first two natural frequencies, **a** amplitude of first mode, **b** amplitude of the second mode

Figure 4, illustrates the frequency resonances in the vicinity of the first two non-dimensional primary resonances. Comparing the amplitude of each individual mode with that of the superharmonic ones depicted in Fig. 2, reveals that the amplitudes in this zone are considerable higher.

**Fig. 5:**
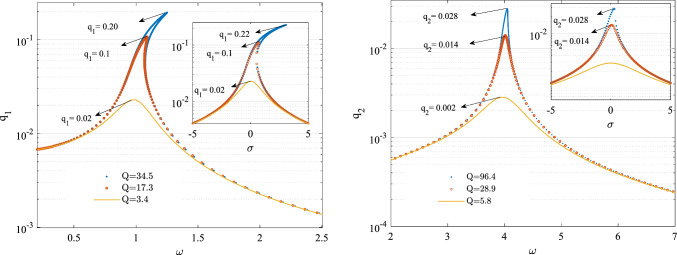
Frequency response curves in the vicinity of the primary resonance based on continuation method and MTS method as inset, **a** amplitude of the first mode, **b** amplitude of the second mode

As shown in Fig. 4, sweeping the frequency in forward direction the generalised amplitude associated with both two modes increase and bend rightward which is due to the hardening nature of the response in the vicinity of both modes. The catastrophic cyclic fold bifurcation (CF) which is immediately followed by a jump, offeres a potential mechnisim of measuring the added mass to the central ring. The results have been derived for four different damping coefficients. As the quality factor increases (indicating a decrease in the damping coefficient), the generalized amplitude also increases. Figure 5 illustrates the frequency response curves associated with the first two modes obtained by both continuation and MTS methods. To ensure that the conditions for the magnitude ordering of the terms in Eq. (9) are met, we have assumed the nondimensionalizing parameters$g={10}^{-5}\text{m} ,\Theta =\frac{\pi }{100} \text{rad}$. This adjustment is intended to reduce the order of magnitude of the nonlinear terms and the excitation terms to the order of$\varepsilon$. The results are obtained for three different damping coefficients asssociated with three different quality factors (*Q*) for each of the modes.

**Fig. 6:**
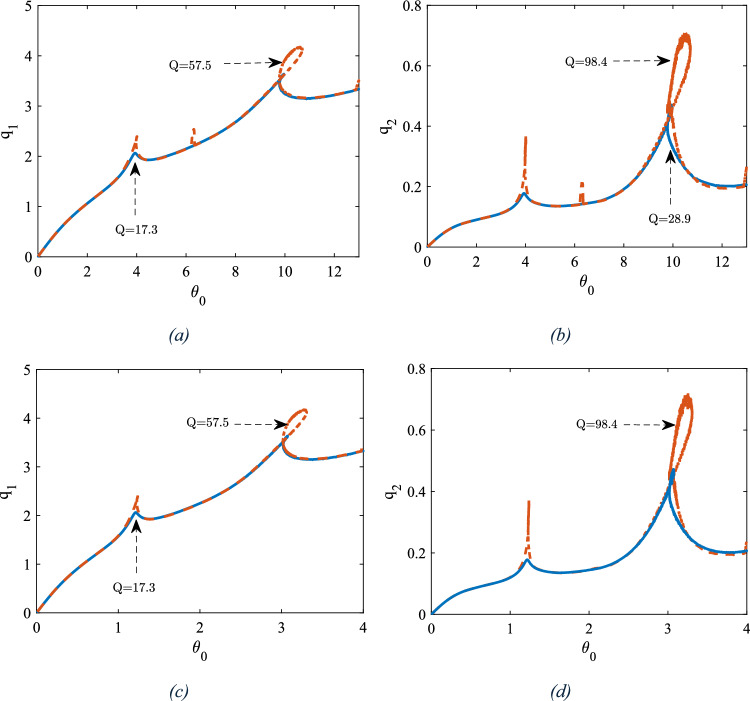
Force response curves of the generalised coordinates for two different excitation frequencies, **a**, **b** $\upomega =0.2$, **c**, **d** $\upomega =0.35$

As illustrated in Fig. 5, the increase in the amplitude of the motion and deviation from the natural frequency no longer keeps either the nonlinear terms or the detuning parameter $\sigma$ in the order of $\varepsilon$. Consequently, the results of the two methods deviate; however, given that the perturbation conditions are satisfied in the vicinity of the frequency ${\omega }_{n}$, there exists substantial agreement between the continuation and MTS methods. Figure 6 shows the force response curve for associated with both of the modes and for two different conditions $\omega =0.2$ and $\omega =0.35$.

**Fig. 7:**
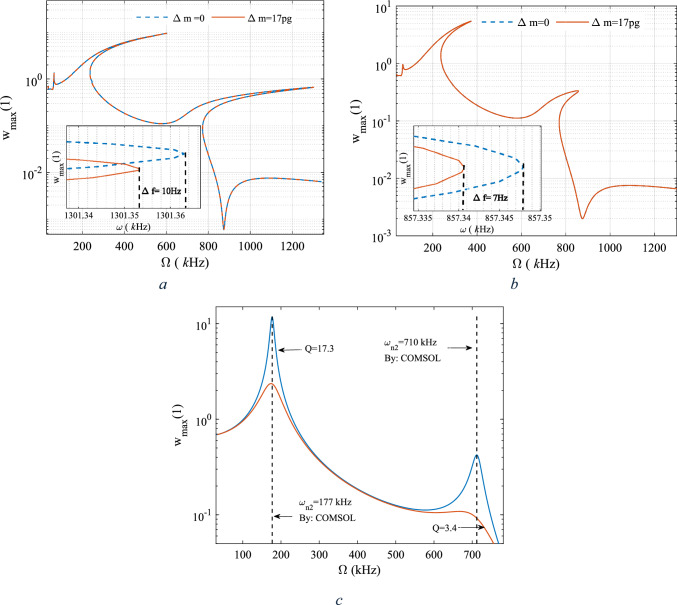
The frequency response curve in the vicinity of the first two natural frequencies, nonlinear analysis **a** Q = 57.5, **b** Q = 17.3, **c** linear analysis

Associated with both excitation frequencies, as the amplitude of the excitation increases, the generalized amplitude of both modes also increases. This behavior is independent of the quality factor. However, as the generalized amplitude of each mode reaches a particular value, the system undergoes a local extremum, after which the amplitude relaxes within a certain range of the force amplitude until the system resumes its increasing behavior. Figure 7a, b depict the frequency response curves with two different masses of the central ring, differing by 17 pg. As shown, with a mass difference of 17 pg, the shift of the peak points of the first two primary resonances are 10 Hz and 7 Hz, for two different quality factors of the first mode respectively; this frequency shift is associated with 0.58 pg/Hz. Figure 7c presents the linear frequency response curves for two different Q factors, alongside the evaluation of the natural frequencies obtained through COMSOL simulations.

As shown, the higher quality factor from which the in-plane resonators benefit leads to better sensitivity of the device to the mass difference of the central ring. The linear frequency response curve (neglecting the nonlinear terms) has been illustrated in Fig. 7c for thwo different Q factors. Finite element analysis is also carried out in COMSOL to verify the linear natural frequencies which there is a very good agreement. Furthermore the frequency shift due to the added mass both in nonlinear and linear modelling is examined. In the nonlinear analysis the sensitivity is 0.58 Hz/ $\text{pg}$ whereas in linear analysis it was 0.5 Hz/ $\text{pg}$ eqivelent to 16% impovement of the sensitivity.

## Conclusion

In this paper, we investigated the dynamics of a micro-ring supported by four clamped beams and subjected to harmonic base excitation. The equations of motion were derived based on the discretization of the Lagrangian, resulting in a reduced-order model. Frequency response curves were obtained using the continuation method and verified using the Method of Multiple Scales (MTS), ensuring the terms were balanced according to the governing equations. Our analysis revealed that as the amplitude of the excitation increases, the results of the perturbation and continuation methods diverge, attributed to the disruption in the balancing of the terms' magnitudes. Frequency response curves were derived for both the super harmonic regime and primary resonances. To identify the sources of specific super harmonic resonances, we examined the frequency content of the response for two different excitation frequencies. This analysis revealed that the amplitude contains odd multiples of the excitation frequency, leading to super harmonic resonance in the regions $\frac{{\omega }_{n}}{2n+1}$, where *n* is a natural number. The effect of the quality factor on the motion amplitudes and the frequency response curves was also examined. To investigate the primary resonance based on the perturbation technique we had to keep the amplitude of the excitation term small enough so that the harmonic excitation force appears not in the very first linear equation, as a result we were unable to compare the two methods for higher amplitudes of the motion. The frequency response curves in the vicinity of primary resonances exhibited a hardening response as they bent rightward on the frequency domain; this was attributed to the type of the supporting beams. It was concluded that the mass difference 17 pg resulted in the measurable frequency shift equal to 10 and 7 Hz in the location of the bifurcation point on the frequency response curves; Besides the nonlinear analysis we carried out linear analysis to compare the effect of nonlinearity on the sensitivity; it was concluded that the sensitivity improves 16% in nonlinear regime. The results of the study are promising in developing and improving the sensitivity and quality factor of MEMS resonators particularly for mass detection applications. Future studies will include the fabrication and testing of the proposed model, during which its mass-sensing capability will be demonstrated.

## Figures and Tables

**Fig. 8:**
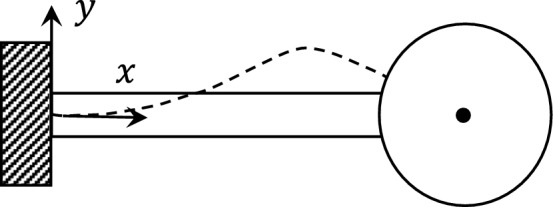
Schematics of a disc with a pin at the centre connected to a clamped beam

## Data Availability

No datasets were generated or analysed during the current study.

## Appendix 1

Assuming a disc with radius *R*, connected to a beam with length *l*, (Fig. 8).

density $\rho$ and young’s modulus *E,* and deflection *w(x,t)* in lateral direction, the kinetic (*T*) and the potential (*U*) energies will be in the following forms:

16 $$ \begin{array}{*{20}l} {T = \frac{{\rho A}}{2}l\left( {\frac{g}{T}} \right)^{2} \smallint _{0}^{1} \dot{w}^{2} ldx + \frac{1}{2}I_{D} \left( {\frac{g}{{lT}}} \right)^{2} } \hfill \\ {\quad \dot{w}_{x} (1,t)^{2} ,U = \frac{{EI}}{2}\left( {\frac{g}{{l^{2} }}} \right)^{2} \int\limits_{0}^{1} {w^{{\prime \prime 2}} ldx} } \hfill \\ \end{array} $$

Non-dimensional parameters $\widehat{x}=x/l , \widehat{w}=w/g , \widehat{t}=t/T$ have been substituted for *x*, *w*, and *t* respectively. For simplicity the hats have been dropped. Here *l*, $g$ and *T* are non-dimensionalising parameters. In Eq. A-1, the rotation of the disc is approximated by the slope of the beam at the connection point to the disc, i.e.,${w}_{x}\left(1,t\right)=\varphi$. Applying integration by parts, the variation of the Hamiltonian, $\delta {\int }_{0}^{t}Hdt=0$ (Nikpourian et al. 2020; Ghavami et al. 2022) has been set to zero.

17 $$ \begin{array}{*{20}l} {\int\limits_{0}^{t} {\left\{ {\int\limits_{0}^{1} {\left( { - \rho Al\left( {\frac{g}{T}} \right)^{2} \ddot{w}\delta w - EI\frac{{g^{2} }}{{l^{3} }}w^{{IV}} \delta w} \right)dx} } \right.} } \hfill \\ {\quad + \left( { - I_{D} \left( {\frac{g}{{Tl}}} \right)^{2} \ddot{w}_{x} \left( {1,t} \right)\delta w_{x} \left( {1,t} \right)} \right.} \hfill \\ {\quad \left. {\left. { - EI\frac{{g^{2} }}{{l^{3} }}w_{{xx}} \left. {\delta w_{x} } \right|_{0}^{1} + EI\frac{{g^{2} }}{{l^{3} }}w_{{xxx}} \left. {\delta w} \right|_{0}^{1} } \right)} \right\}dt} \hfill \\ {\quad = 0} \hfill \\ \end{array} $$

Assuming ${T}^{2}=\frac{\rho A{l}^{4}}{EI}$ the linear motion equation and the associated boundary conditions reduce to:

$$\ddot{w}+{w}^{IV}=0$$

18 $$ \begin{array}{*{20}l} { - I_{D} \left( {\frac{g}{{Tl}}} \right)^{2} \ddot{w}_{x} \left( {1,t} \right)\delta w_{x} \left( {1,t} \right) - EI\frac{{g^{2} }}{{l^{3} }}w_{{xx}} \left( {1,t} \right)\delta w_{x} \left( {1,t} \right)} \hfill \\ {\quad + EI\frac{{g^{2} }}{{l^{3} }}w_{{xxx}} \left( {1,t} \right)\delta w\left( {1,t} \right) + EI\frac{{g^{2} }}{{l^{3} }}w_{{xx}} \left( {0,t} \right)\delta w_{x} \left( {0,t} \right)} \hfill \\ {\quad - EI\frac{{g^{2} }}{{l^{3} }}w_{{xxx}} \left( {0,t} \right)\delta w\left( {0,t} \right) = 0} \hfill \\ \end{array} $$

With the assumption $\delta w\left(1,t\right)=-\frac{R}{l}\delta {w}_{x}(1,t)$, the boundary condition terms in Eq. (18) reduce to:

19 $${I}_{D}{\left(\frac{g}{Tl}\right)}^{2}{\ddot{w}}_{x}\left(1,t\right)\frac{l}{R}+EI\frac{{g}^{2}}{{l}^{3}}{w}_{xxx}\left(1,t\right)+EI\frac{{g}^{2}}{{l}^{3}}{w}_{xx}\left(1,t\right)\frac{l}{R}=0$$

$$w\left(0,t\right)=0, {w}_{x}\left(0,t\right)=0, w\left(1,t\right)=-\frac{R}{l}$$

The first two shape functions which satisfy the boundary conditions specified in Eq. (19) have been derived as follows:

20 $$ \begin{array}{*{20}l} {\psi _{1} \left( x \right) = 2.8743{\text{sin}}\left( {1.8958x} \right) - 2.0588{\text{cos}}\left( {1.8958x} \right)} \hfill \\ {\quad - 2.8743{\text{sinh}}\left( {1.8958x} \right) + 2.0588{\text{cosh}}\left( {1.8958x} \right)} \hfill \\ \end{array} $$

$$ \begin{array}{*{20}l} {\psi _{1} \left( x \right) = 1.0015{\text{sin}}\left( {4.8111x} \right) - 1.0177{\text{cos}}\left( {4.8111x} \right)} \hfill \\ {\quad - 1.0015{\text{sinh}}\left( {4.8111x} \right) + 1.0177{\text{cosh}}\left( {1.8958x} \right)} \hfill \\ \end{array} $$

## Appendix 2

The coefficients of the terms in Eq. (8) are as follows:

**Table:**

${M}_{1}=2{{\alpha }_{1}}_{11}+2{{\alpha }_{2}}_{11}$	${M}_{2}={{\alpha }_{1}}_{21}+{{\alpha }_{2}}_{21}+{{\alpha }_{1}}_{12}+{{\alpha }_{2}}_{12}$
${Kl}_{1}=2{{\beta }_{1}}_{11}-2{\Gamma }_{{1}_{11}}$	${Kl}_{2}={{\beta }_{1}}_{21}+{{\beta }_{1}}_{12}-{\Gamma }_{{1}_{21}}-{\Gamma }_{{1}_{12}}$
${Kn}_{1}=4{{\beta }_{2}}_{1111}$	${Kn}_{2}={{\beta }_{2}}_{2221}+{{\beta }_{2}}_{2212}+{{\beta }_{2}}_{2122}+{{\beta }_{2}}_{1222}$
${C}_{1}=2{\gamma }_{11}$	${Kg}_{1}=2{{\beta }_{2}}_{1221}+2{{\beta }_{2}}_{2112}+2{{\beta }_{2}}_{1212}+2{{\beta }_{2}}_{2211}+2{{\beta }_{2}}_{1122}+2{{\beta }_{2}}_{2121}$
$${C}_{2}={\gamma }_{12}+{\gamma }_{21}$$	
${F}_{1}=-{\dot{\Gamma }}_{{2}_{1}}-{\dot{\Gamma }}_{{3}_{1}}$	${Kg}_{2}=3{{\beta }_{2}}_{1112}+3{{\beta }_{2}}_{2111}+3{{\beta }_{2}}_{1211}+3{{\beta }_{2}}_{1121}$
$${M}_{3}=2{{\alpha }_{1}}_{22}+2{{\alpha }_{2}}_{22}$$	
${Kl}_{3}=2{{\beta }_{1}}_{22}-2{\Gamma }_{{1}_{22}}$	${Kn}_{4}=4{{\beta }_{2}}_{2222}$
$${Kn}_{3}={{\beta }_{2}}_{1112}+{{\beta }_{2}}_{2111}+{{\beta }_{2}}_{1211}+{{\beta }_{2}}_{1121}$$	
${C}_{3}=2{\gamma }_{22}$	${Kg}_{3}=3{{\beta }_{2}}_{2122}+3{{\beta }_{2}}_{1222}+3{{\beta }_{2}}_{2221}+3{{\beta }_{2}}_{2212}$ ${Kg}_{4}=2{{\beta }_{2}}_{1212}+2{{\beta }_{2}}_{2121}+2{{\beta }_{2}}_{1212}+2{{\beta }_{2}}_{2211}+2{{\beta }_{2}}_{1122}+2{{\beta }_{2}}_{2112}$
$${F}_{2}=-{\dot{\Gamma }}_{{2}_{2}}-{\dot{\Gamma }}_{{3}_{2}}$$	

where:

**Table:**

${{\alpha }_{1}}_{ij}=\frac{1}{2}{n}_{s}{\left(\rho A\right)}_{s}\frac{{g}^{2}{l}_{s}}{{T}^{2}}{\int }_{0}^{1}{\psi }_{{s}_{i}}\left({x}_{s}\right){\psi }_{{s}_{j}}\left({x}_{s}\right)d{x}_{s}$	${\gamma }_{ij}=\frac{1}{2}{n}_{s}{c}_{s}\frac{{g}^{2}{l}_{s}}{{T}^{2}}{\int }_{0}^{1}{\psi }_{{s}_{i}}\left({x}_{s}\right){\psi }_{{s}_{j}}\left({x}_{s}\right)d{x}_{s}$
$\alpha_{2ij} = \frac{1}{2}I_{D} \frac{{g^{2} }}{{l_{s}^{2} T^{2} }}\psi_{{s_{i} }}^{\prime } \left( 1 \right)\psi_{{s_{j} }}^{\prime } \left( 1 \right)$	${\Gamma }_{{1}_{ij}}=\frac{1}{2}{n}_{s}{\left(\rho A\right)}_{s}{\dot{\theta }}^{2}\frac{{\Theta }^{2}{g}^{2}{l}_{s}}{{T}^{2}}{\int }_{0}^{1}{\psi }_{{s}_{i}}({x}_{s}){\psi }_{{s}_{j}}({x}_{s})d{x}_{s}$
$$\beta_{{1_{ij} }} = \frac{1}{2}n_{s} \left( {EI} \right)_{s} \frac{{g^{2} }}{{l_{s}^{3} }}\mathop \smallint \limits_{0}^{1} \psi_{{s_{i} }}^{\prime \prime } \left( {x_{s} } \right)\psi_{{s_{j} }}^{\prime \prime } \left( {x_{s} } \right)dx_{s}$$	
${\Gamma }_{{2}_{i}}=-{n}_{s}{\left(\rho A\right)}_{s}\dot{\theta }\frac{\Theta g{l}_{s}}{{T}^{2}}{\int }_{0}^{1}\left({R}_{o}-{l}_{s}{x}_{s}\right){\psi }_{{s}_{i}}({x}_{s})d{x}_{s}$	$\Gamma_{3i} = I_{D} \dot{\theta }\frac{\Theta g}{{T^{2} l_{s} }}\psi_{{s_{i} }}^{\prime } \left( 1 \right)$
$$\beta_{{2_{ijko} }} = \frac{EA}{8}\frac{{g^{4} }}{{l_{s}^{3} }}n_{s} \mathop \smallint \limits_{0}^{1} \psi_{{s_{i} }}^{\prime } \left( {x_{s} } \right)\psi_{{s_{j} }}^{\prime } \left( {x_{s} } \right)dx_{s} \mathop \smallint \limits_{0}^{1} \psi_{{s_{k} }}^{\prime } \left( {x_{s} } \right)\psi_{{s_{o} }}^{\prime } \left( {x_{s} } \right)dx_{s}$$	
